# Perinatal complications and neonatal outcomes in *in vitro* fertilization/intracytoplasmic sperm injection: a propensity score matching cohort study

**DOI:** 10.3389/fendo.2024.1405550

**Published:** 2024-07-18

**Authors:** Ying Chen, Mengjie Zhang, Yumei Gao, Mingming Li, Wenjun Zheng, Xueyan Guo, Fei Li

**Affiliations:** ^1^ Center for Reproductive Medicine, The First People’s Hospital of Shangqiu, Clinical College affiliated to XuZhou Medical University, Shangqiu, Henan, China; ^2^ Center for Reproductive Medicine, The First Affiliated Hospital of Xinjiang Medical University, Urumchi, Xinjiang, China; ^3^ Department of Gynaecology, Graduate School of Zhengzhou University, Zhengzhou, Henan, China

**Keywords:** *in vitro* fertilization/intracytoplasmic sperm injection, natural pregnancies, perinatal complications, neonatal outcomes, propensity score matching

## Abstract

**Background:**

The utilization of *in vitro* fertilization/intracytoplasmic sperm injection (IVF/ICSI) has witnessed a significant increase in recent years. However, the comparative perinatal and neonatal outcomes compared to natural pregnancies are unclear. This study aims to compare the outcomes of pregnancies from IVF and ICSI with natural pregnancies.

**Methods:**

This retrospective, propensity score-matched cohort study was conducted at the First People’s Hospital of Shangqiu and The First Affiliated Hospital of Xinjiang Medical University, involving 5,628 patients from February 2019 to December 2022. It compared pregnancies achieved through IVF/ICSI with those conceived naturally. The primary outcomes assessed were perinatal complications and neonatal health parameters. Propensity score matching and multivariate logistic regression analysis were employed to adjust for potential confounders and identify independent associations.

**Results:**

After propensity score matching, the IVF/ICSI group demonstrated significantly higher rates of placental adherence (12.1% vs. 7.4%, *p* < 0.001) and postpartum hemorrhage (11.1% vs. 7.6%, *p* = 0.002) compared to the NP group. Neonates in the IVF/ICSI group had a lower gestational age (38.21 ± 2.12 weeks vs. 38.63 ± 2.29 weeks, *p* < 0.001), reduced birth weight (3159.42 ± 722.75 g vs. 3211.31 ± 624.42 g, *p* = 0.032), and an increased preterm delivery rate (11.2% vs. 8.9%, *p* = 0.017). Multivariate analysis further confirmed these findings, highlighting the independent associations between IVF/ICSI and these adverse outcomes.

**Conclusion:**

This study suggests a potential correlation between the use of IVF/ICSI and unfavorable perinatal and neonatal outcomes. These findings underscore the critical need for ongoing monitoring and research efforts to enhance the safety and effectiveness of these reproductive technologies.

## Introduction

In recent decades, the utilization of assisted reproductive technology (ART), specifically *in vitro* fertilization/intracytoplasmic sperm injection (IVF/ICSI), has experienced a significant surge globally (1, 2). These techniques have provided invaluable opportunities for myriad infertile couples seeking to conceive. However, with the increasing adoption of these methods, concerns have emerged regarding their possible effects on perinatal complications and neonatal outcomes, particularly in contrast to outcomes observed in natural pregnancies (NP) (3–5). NP is characterized by the unassisted union of sperm and egg within the female body through sexual intercourse, culminating in the development of a fertilized egg without the need for medical intervention or fertility treatments.

Prior research on the perinatal and neonatal outcomes of pregnancies achieved through IVF/ICSI has produced equivocal results (6, 7). Some studies suggest an increased risk for specific complications, such as gestational hypertension, gestational diabetes, and preterm birth (8, 9). Lei et al (10) discovered a significant correlation between ART and an elevated occurrence of gestational diabetes mellitus and intrahepatic cholestasis of pregnancy, in contrast to pregnancies resulting from natural conception. Bianchi et al’s study revealed that gestational diabetes occurs in approximately 50% of women undergoing ART treatment (11). Conversely, other studies have not identified any noteworthy disparities between IVF/ICSI and NP (12–14). One study showed that newborns born after IVF/ICSI following testicular sperm aspiration had similar neonatal outcomes to those born with natural conception (15). Joshi et al (16) conducted an analysis of data from the National Assisted Reproductive Technology Surveillance System, which indicated a rise in favorable perinatal outcomes for live births resulting from assisted reproductive technology. These discrepancies may be ascribed to diverse factors, such as variations in research methodologies, sample sizes, and the incorporation of confounding variables. In light of these uncertainties, it is imperative to possess a thorough comprehension of the perinatal and neonatal consequences linked to pregnancies resulting from IVF/ICSI. This knowledge is vital for providing appropriate guidance to patients, formulating clinical guidelines, and enhancing care strategies.

Consequently, this retrospective cohort study utilizing propensity score matching (PSM) was undertaken to examine and contrast the perinatal complications and neonatal outcomes of pregnancies resulting from IVF/ICSI in comparison to NP. The study places particular emphasis on mitigating any initial disparities between the two groups. By elucidating the autonomous associations between IVF/ICSI and these outcomes, the study endeavors to augment the current knowledge base and provide guidance for clinical practice in this dynamically progressing domain.

## Materials and methods

### Study design and population

**Figure 1:**
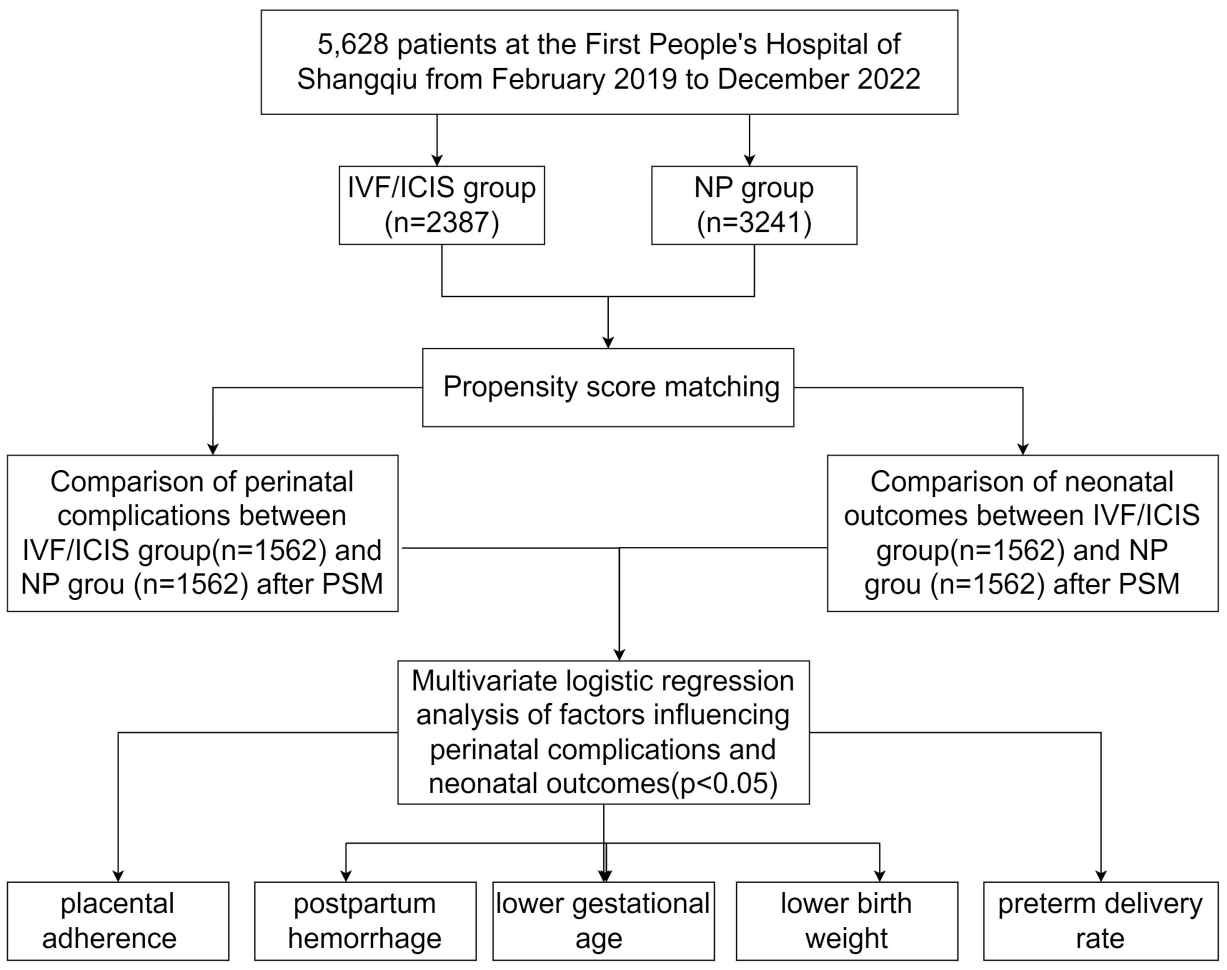
The technology roadmap.

We conducted a retrospective propensity score-matched cohort study to compare the perinatal complications and neonatal outcomes in pregnancies achieved through IVF/ICSI versus natural pregnancies. The study population comprised patients who underwent IVF/ICSI treatment at the First People’s Hospital of Shangqiu and The First Affiliated Hospital of Xinjiang Medical University between February 2019 and December 2022. A total of 2387 patients were included in the IVF/ICSI group. For the NP group, we randomly selected 3241 patients from the hospital’s obstetrics and gynecology database, who delivered during the same period and met the inclusion criteria. The study protocol was approved by the ethics committee of the First People’s Hospital of Shangqiu (No: HS20230334). All procedures involving human participants were conducted in accordance with the ethical standards of the institutional and/or national research committee and with the 1964 Helsinki declaration and its later amendments or comparable ethical standards. Informed consent was waived due to the retrospective nature of the study. The technology roadmap is shown in **Figure 1**.

Inclusion criteria: Individuals aged between 18 to 45 years, possessing comprehensive medical records and follow-up information. Exclusion criteria: Pregnant women with uterine malformations or anomalies, history of ovarian or pelvic surgeries, and those presenting with severe underlying conditions or complications.

### Ovulation induction protocols for IVF/ICSI

Long-acting follicular phase protocol, which involves intramuscular injection of gonadotropin-releasing hormone agonist (GnRH-a, Diphereline, 3.75mg/vial, Ipsen Pharma, France) 3.75mg on days 2-4 of the menstrual cycle. After meeting the pituitary downregulation criteria, gonadotropin (Gn, Lishenbao, 75U/vial, Zhuhai Livzon, 150-300U/d; or Gonal-F, 75U/vial, Merck Serono, Germany, 100-300U/d; or Puregon, 100U/vial, MSD, Germany, 100-300U/d) is administered for ovulation induction; Long-acting mid-luteal phase protocol, which involves subcutaneous injection of GnRH-a (Diphereline, 3.75mg/vial, Ipsen Pharma, France) 1.5-1.875mg on days 20-23 of the menstrual cycle. After 16-20 days of injection and meeting the pituitary downregulation criteria, Gn is administered for ovulation induction based on the patient’s age, body mass index (BMI), and ovarian reserve; Antagonist protocol, which involves the administration of Gn for ovulation induction on days 2-4 of the menstrual cycle based on the patient’s age, BMI, and ovarian reserve. When the dominant follicle diameter reaches more than 14mm and estradiol is greater than 300ng/L, gonadotropin-releasing hormone antagonist (Cetrotide, 0.25mg/vial, Merck Serono, Switzerland) is simultaneously administered subcutaneously at a dose of 0.25mg/d. The dosage of Gn is adjusted according to ovarian responsiveness and hormone levels during the medication process; When the dominant follicle diameter reaches 20mm or at least 2-3 follicles reach a diameter of 18mm in the above three protocols, hCG (Ovidrel, 250μg/vial, Merck Serono, Germany) 250μg trigger shot is administered, followed by oocyte retrieval under transvaginal ultrasound guidance 36-38 hours later.

### Propensity score matching

To minimize potential baseline disparities between the IVF/ICSI and NP groups, we employed propensity score matching. The propensity score was estimated using a logistic regression model, which included potential confounders such as age, body mass index (BMI), parity, smoking status, and history of chronic diseases. Patients in the IVF/ICSI group were then matched 1:1 with patients in the NP group based on the closest propensity score, within a caliper width of 0.2 standard deviations (17, 18).

### Data collection and outcome measures

Data on perinatal complications and neonatal outcomes were extracted from the patients’ medical records. Perinatal complications included gestational hypertension, gestational diabetes, preeclampsia, intrahepatic cholestasis, placental abruption, placental adherence, postpartum hemorrhage, polyhydramnios, oligohydramnios, weight of placenta. Neonatal outcomes comprised of gestational age, birth weight, proportion of male newborns, birth height, and preterm delivery rate. All outcomes were defined according to standard clinical criteria.

### Statistical analysis

Statistical analysis for this study was conducted using SPSS version 26.0 (IBM, Chicago, USA). Dichotomous variables were expressed as proportions or percentages (%), while continuously distributed variables with a normal distribution were expressed as mean ± standard deviation. Comparisons between two independent samples for dichotomous variables were analyzed using the chi-square test or Fisher’s exact test, as appropriate. For comparisons between two groups of independent samples with normally distributed and homoscedastic continuous variables, the independent samples t-test was used.

Based on the delivery dates of mothers in the IVF/ICSI group, mothers in Group NP who delivered on the same day were numbered. Subsequently, mothers in the NP group were randomly selected using computer software. The PSM method was then applied to perform 1:1 matching for baseline data that showed statistically significant differences between the two groups. Variables included in the matching process were female age, BMI, diabetes history, hypertension History, maternal education and delivery mode.

Multivariate logistic regression analysis was employed to identify risk factors for perinatal complications and neonatal outcomes. The results for dichotomous variables were expressed using odds ratio (OR) with their corresponding 95% confidence intervals (CIs), while the results for continuous variables were presented as mean differences (MDs) with their 95% CIs. *p*< 0.05 was considered statistically significant.

## Results

A total of 5,628 subjects were included in this study, prior to matching, significant differences were observed in baseline characteristics such as female age, male age, number of births, BMI, and educational level. However, after propensity score matching, these differences were effectively eliminated, ensuring a more accurate comparison between the two groups (**Table 1**).

**Table 1:** Comparison of baseline characteristics between the two groups.

Projects	Before propensity score matching			After propensity score matching		
	NP group (n=3241)	IVF/ICIS group (n=2387)	*p* value	NP group (n=1562)	IVF/ICIS group (n=1562)	*p* value
Female Age (years)	25.9 ± 2.3	29.7 ± 2.5	<0.001	27.6 ± 2.3	27.8 ± 2.5	0.120
Male Age (years)	27.9 ± 2.2	30.7 ± 2.3	<0.001	28.6 ± 2.5	28.9 ± 2.7	0.264
**Number of births (n)**						0.850
0	1892 (58.4%)	1623 (67.9%)	<0.001	942 (60.3%)	937 (60.0%)	
1	1023 (31.6%)	582 (24.4%)		475 (30.4%)	487 (31.2%)	
≥2	326 (10.1%)	182 (7.6%)		145 (9.3%)	138 (8.8%)	
**BMI (**kg/m^2)						0.602
<18.5	311 (9.6%)	159 (6.7%)	<0.001	162 (10.4%)	154 (9.8%)	
18.5-23.9	2061 (63.6%)	1361 (57.1%)		802 (51.3%)	818 (52.4%)	
24.0-27.9	672 (20.7%)	470 (19.7%)		410 (26.2%)	398 (25.5%)	
≥28	197 (6.1%)	397 (16.6%)		188 (12.1%)	192 (12.3%)	
**Smoking (%)**						0.857
Yes	33 (1.0%)	25 (1.0%)	0.890	16 (1.0%)	15 (1.0%)	
No	3208 (98.9%)	2362 (99.0%)		1546 (99.0%)	1547 (99.0%)	
**Diabetes history (%)**						0.883
Yes	44 (1.4%)	49 (2.1%)	0.043	24 (1.5%)	23 (1.5%)	
No	3197 (98.6%)	2338 (97.9%)		1538 (98.5%)	1539 (98.5%)	
**Hypertension History (%)**						0.872
Yes	38 (1.2%)	31 (1.3%)	0.762	19 (1.2%)	20 (1.3%)	
No	3203 (98.8%)	2356 (98.7%)		1543 (98.8%)	1542 (98.7%)	
**Maternal education (%)**						0.850
≤Secondary school	892 (27.5%)	1028 (43.1%)	<0.001	432 (27.6%)	427 (27.3%)	
College graduate	1356 (41.8%)	1036 (43.4%)		690 (44.2%)	700 (44.8%)	
Post-graduate	493 (15.2%)	323 (13.5%)		240 (15.4%)	239 (15.3%)	
**Delivery mode (%)**						0.747
Vaginal delivery	1752 (53.9%)	1529 (64.1%)	<0.001	812 (52.0%)	803 (51.4%)	
Cesarean delivery	1489 (46.1%)	858 (35.9%)		750 (48.0%)	759 (48.6%)	

Data are shown as means ± SD or percentages. NP: Natural pregnancy; IVF/ICIS: in vitro fertilization and intracytoplasmic sperm injection; BMI, body mass index.

In terms of perinatal complications, the IVF/ICSI group exhibited a notably elevated incidence of gestational hypertension (5.7% vs. 3.4%, *p* = 0.012), placental adherence (12.1% vs. 7.4%, *p* < 0.001), and postpartum hemorrhage (11.1% vs. 7.6%, *p* = 0.002) in comparison to the NP group. Although the rates of gestational diabetes, preeclampsia, intrahepatic cholestasis, placental abruption, premature rupture of membranes, polyhydramnios, oligohydramnios, and placental weight were comparable between the groups, a discernible trend toward elevated rates of specific complications was evident in the IVF/ICSI group (**Table 2**).

**Table 2:** Comparison of perinatal complications between IVF/ICIS group and NP group after propensity score matching.

Projects	After propensity score matching		
	NP group (n=1562)	IVF/ICIS group (n=1562)	*p* value
Gestational hypertension (%)	53 (3.4%)	89 (5.7%)	0.012
gestational diabetes (%)	178 (11.4%)	204 (13.1%)	0.124
**Preeclampsia (%)**			0.249
Mild	32 (2.1%)	37 (2.4%)	
Severe	62 (4.0%)	73 (4.7%)	
Intrahepatic cholestasis (%)	94 (6.0%)	110 (7.0%)	0.963
Placental abruption (%)	79 (5.1%)	80 (5.1%)	0.245
**Placenta Previa (%)**			0.002
Complete	14 (0.9%)	28 (1.8%)	
Partial	32 (2.1%)	49 (3.1%)	
Marginal	55 (3.5%)	76 (4.9%)	
Premature rupture of membranes (%)	163 (10.4%)	171 (10.9%)	0.623
Placental adherence (%)	116 (7.4%)	189 (12.1%)	<0.001
Postpartum hemorrhage (%)	119 (7.6%)	173 (11.1%)	0.002
Polyhydramnios (%)	62 (4.0%)	77 (4.9%)	0.249
Oligohydramnios (%)	103 (6.6%)	114 (7.3%)	0.459
Weight of placenta (g)	521.92 ± 120.23	562.21 ± 138.13	<0.001

Data are shown as means ± SD or percentages. NP: Natural pregnancy; IVF/ICIS: in vitro fertilization and intracytoplasmic sperm injection.

With regard to neonatal outcomes, the IVF/ICSI group demonstrated a markedly reduced gestational age (38.21 ± 2.12 weeks vs. 38.63 ± 2.29 weeks, *p* < 0.001) and birth weight (3159.42 ± 722.75 g vs. 3211.31 ± 624.42 g, *p* = 0.032) in comparison to the NP group. Additionally, the IVF/ICSI group had a higher percentage of male newborns (55.4% vs. 51.3%, *p* = 0.028) and a higher preterm delivery rate (11.2% vs. 8.9%, *p* = 0.017). No significant differences were found in birth height, the incidence of macrosomia, 1-minute Apgar score, 5-minute Apgar score, or the incidence of birth defects between the two groups (**Table 3**).

**Table 3:** Comparison of neonatal outcomes between IVF/ICIS group and NP group after propensity score matching.

Projects	After propensity score matching		
	NP group (n=1562)	IVF/ICIS group (n=1562)	*p* value
Gestational age(week)	38.63 ± 2.29	38.21 ± 2.12	<0.001
Birth weight(g)	3211.31 ± 624.42	3159.42 ± 722.75	0.032
Proportion of male newborns (%)	802 (51.3%)	865 (55.4%)	0.028
Preterm delivery rate (%)	139 (8.9%)	175 (11.2%)	0.017
Birth height (cm)	50.40 ± 4.11	50.19 ± 3.93	0.143
Incidence of macrosomia (%)	84 (5.4%)	79 (5.1%)	0.603
1 min Apgar score ≤ 7 (%)	76 (4.9%)	88 (5.6%)	0.291
5 min Apgar score ≤ 7 (%)	27 (1.7%)	32 (2.0%)	0.469
Incidence of birth defects (%)	23 (1.5%)	35 (2.2%)	0.112

Data are shown as means ± SD or percentages. NP: Natural pregnancy; IVF/ICIS: in vitro fertilization and intracytoplasmic sperm injection.

Multivariate logistic regression analysis was performed to further explore the factors influencing perinatal complications and neonatal outcomes. After adjusting for potential confounders, the results showed that placental adherence (odds ratio [OR] = 1.241, 95% confidence interval [CI] = 1.072–1.512, *p* < 0.001) and postpartum hemorrhage (OR = 1.125, 95% CI = 1.041–1.315, *p* = 0.031) were independently associated with IVF/ICSI pregnancies. Gestational age (OR = 0.318, 95% CI = 0.231–0.449, *p* < 0.001) and birth weight (OR = 0.842, 95% CI = 0.671–0.970, *p* = 0.029) were also significantly associated with the method of conception, with lower values observed in the IVF/ICSI group (**Table 4**).

**Table 4:** Multivariate logistic regression analysis of factors influencing perinatal complications and neonatal outcomes.

Factors	Multivariate Logistic				
	NP group (n=1562)	IVF/ICIS group (n=1562)	Exp (B)	95% C.I	*p* value
Gestational hypertension (%)	53 (3.4%)	89 (5.7%)	1.062	0.925~1.152	0.410
Placenta Previa (%)					
Complete	14 (0.9%)	28 (1.8%)	1.296	0.828~1.923	0.291
Partial	32 (2.1%)	49 (3.1%)	1.424	0.982~2.015	0.090
Marginal	55 (3.5%)	76 (4.9%)	1.112	0.880~1.429	0.398
Placental adherence (%)	116 (7.4%)	189 (12.1%)	1.241	1.072~1.512	<0.001
Postpartum hemorrhage (%)	119 (7.6%)	173 (11.1%)	1.125	1.041~1.315	0.031
Gestational age(week)	38.63 ± 2.29	38.21 ± 2.12	0.318	0.231~0.449	<0.001
Birth weight(g)	3211.31 ± 624.42	3159.42 ± 722.75	0.842	0.671~0.970	0.029
Proportion of male newborns (%)	802 (51.3%)	865 (55.4%)	1.065	0.721~1.293	0.782
Preterm delivery rate (%)	139 (8.9%)	175 (11.2%)	1.234	1.029~1.403	0.023

Data are shown as means ± SD or percentages. NP: Natural pregnancy; IVF/ICIS: in vitro fertilization and intracytoplasmic sperm injection.

## Discussion

This study provides a comprehensive analysis of perinatal complications and neonatal outcomes in pregnancies resulting from IVF/ICSI and natural pregnancy. By employing propensity score matching to address initial differences, our findings reveal that the IVF/ICSI group demonstrates increased occurrences of gestational hypertension, placental adherence, and postpartum hemorrhage. Additionally, this group exhibits a shorter gestational age, lower birth weight, a higher proportion of male infants, and a higher rate of preterm deliveries in terms of neonatal outcomes, and the findings from multivariate analysis provide additional support for the independent relationships between IVF/ICSI and the aforementioned adverse outcomes. These observations emphasize the importance of increased vigilance and effective management of perinatal complications in pregnancies resulting from IVF/ICSI, especially considering the growing global utilization of Assisted Reproductive Technologies.

The higher incidence rates of gestational hypertension, placental adherence, and postpartum hemorrhage observed in the IVF/ICSI cohort align with previous research findings. For instance, a meta-analysis conducted by Thomopoulos et al. reported a higher incidence of hypertensive disorders in IVF pregnancies compared to natural conceptions (19). Similarly, a recent retrospective cohort study by He et al. analyzing data from 10 reproductive centers in Shanghai between 2013 and 2018, encompassing 5,960 cases of ART singleton live births and 8,005 cases of NP singleton live births, concluded that ART assistance in pregnancy increased the risk of premature delivery and other pregnancy complications (20). Wang et al. reported that the excess risk of gestational hypertension and preeclampsia due to ART treatment, the study found that ART mothers had a 17% higher likelihood of experiencing gestational hypertension and preeclampsia compared to non-ART mothers, with an odds ratio of 1.17 (95% confidence interval, 1.10-1.24). After stratification by plurality, the difference in gestational hypertension and preeclampsia rates between ART and non-ART mothers was not statistically significant (21), this consistency reinforces the validity of our findings. The observed increase in placental adherence in the IVF/ICSI group could plausibly be attributed to the altered hormonal and immunological milieu characteristic of these pregnancies, which may implicate placental development and function (22–24). Moreover, the higher incidence of postpartum hemorrhage in this context might be linked to the increased rates of placental adherence, given that adherence issues can lead to retained placenta and subsequent bleeding (25, 26).

The present study observed a decrease in gestational age, a decrease in birth weight, and an increase in the rate of preterm deliveries in pregnancies achieved through IVF/ICSI. A meta-analysis conducted by Pandey et al. demonstrated that neonates born following IVF/ICSI exhibited a higher risk of preterm birth and low birth weight compared to infants conceived naturally (27). Similarly, the study by Hamilton et al. reported a lower gestational age and a higher occurrence of preterm delivery in pregnancies resulting from assisted reproductive technology. These findings are consistent with previous literature reports (28). These findings align with previous reports in the literature and the underlying mechanisms of these adverse outcomes are multifaceted, encompassing both embryonic and maternal factors (29, 30). Embryonic factors may stem from the manipulation of embryos during the IVF/ICSI procedure, potentially altering their subsequent development. On the other hand, maternal factors may comprise underlying infertility, advanced age, and the utilization of ovarian stimulation drugs, all of which may contribute to the disparities observed in neonatal outcomes (11, 31, 32). Moreover, it is essential to emphasize an additional pivotal aspect: the augmented significance attributed to embryos acquired through IVF/ICSI by patients. This heightened value may result in a greater inclination towards elective cesarean sections or induced labor, prompted by apprehensions regarding fetal distress or associated hazards. Consequently, this propensity can ultimately lead to an increased probability of diminished gestational age or premature delivery. Our study uncovers an intriguing phenomenon wherein the proportion of male infants marginally exceeds that of female infants. Similarly, Tan et al. reported a higher male-to-female ratio among offspring conceived through IVF/ICSI, which may be linked to inadequate X-chromosome inactivation (Lyonization process) in female embryos, a process in which one of the two X chromosomes in female mammalian cells becomes inactive (33). Nonetheless, the increased ratio of male infants observed in IVF/ICSI procedures could also be influenced by a variety of unknown factors, including social and technical considerations, requiring further exploration in the future.

The study’s strengths lie in the meticulous implementation of propensity score matching, which effectively created balanced groups and minimized the impact of confounding variables (34, 35). Additionally, the study’s robust sample size and comprehensive evaluation of perinatal complications and neonatal outcomes enhance its credibility. Nevertheless, despite these strengths, some limitations remain. Firstly, the retrospective design of our study limits the ability to establish definitive causal relationships between IVF/ICSI and the observed effects. Secondly, although propensity score matching was utilized, it is not possible to completely eliminate all potential confounding variables with absolute certainty. Lastly, the absence of data on specific complications during pregnancy and childbirth, which are known to have a substantial impact on outcomes, poses limitations to our analysis.

In summary, our research, employing PSM to address initial differences, has revealed notable disparities in perinatal complications and neonatal outcomes between pregnancies achieved through IVF/ICSI and natural pregnancies. Specifically, the IVF/ICSI cohort exhibited elevated rates of gestational hypertension, placental adherence, and postpartum hemorrhage. Regarding neonatal outcomes, the IVF/ICSI group demonstrated a reduced gestational age, lower birth weight, and an increased preterm delivery rate. These findings emphasize the necessity of ongoing monitoring and dedicated research endeavors in this field to enhance outcomes for both mothers and newborns.

## Data availability statement

The raw data supporting the conclusions of this article will be made available by the authors, without undue reservation.

## Ethics statement

This study was conducted in strict compliance with the relevant requirements of Declaration of Helsinki of World Medical Association and approved by the Ethics Committee of the First People’s Hospital of Shangqiu (No: HS20230334). The studies were conducted in accordance with the local legislation and institutional requirements. The ethics committee/institutional review board waived the requirement of written informed consent for participation from the participants or the participants’ legal guardians/next of kin due to the retrospective nature of the study. 

## Author contributions

YC: Conceptualization, Data curation, Formal analysis, Investigation, Methodology, Project administration, Resources, Software, Supervision, Validation, Visualization, Writing – original draft, Writing – review & editing. MZ: Data curation, Formal analysis, Investigation, Software, Writing – review & editing. YG: Data curation, Formal analysis, Project administration, Resources, Writing – review & editing. ML: Data curation, Project administration, Writing – review & editing. WZ: Methodology, Project administration, Software, Writing – review & editing. XG: Software, Writing – original draft. FL: Conceptualization, Data curation, Funding acquisition, Investigation, Methodology, Project administration, Resources, Software, Supervision, Validation, Writing – original draft, Writing – review & editing.
